# Multiple window spatial registration error of a gamma camera: ^133^Ba point source as a replacement of the NEMA procedure

**DOI:** 10.1186/1756-6649-8-6

**Published:** 2008-12-09

**Authors:** Helmar Bergmann, Gregory Minear, Maria Raith, Peter M Schaffarich

**Affiliations:** 1 Center for Biomedical Engineering and Physics, Vienna Medical University, Waehringer Guertel 18 – 20, A-1090 Vienna, Austria; 2 Dept of Nuclear Medicine, Landesklinikum, Propst-Fuehrer-Straße 4, A-3100 St. Pölten, Lower Austria; 3 Clinic of Nuclear Medicine, Vienna Medical University, Waehringer Guertel 18 – 20, A-1090 Vienna, Austria

## Abstract

**Background:**

The accuracy of multiple window spatial resolution characterises the performance of a gamma camera for dual isotope imaging. In the present study we investigate an alternative method to the standard NEMA procedure for measuring this performance parameter.

**Methods:**

A long-lived ^133^Ba point source with gamma energies close to ^67^Ga and a single bore lead collimator were used to measure the multiple window spatial registration error. Calculation of the positions of the point source in the images used the NEMA algorithm. The results were validated against the values obtained by the standard NEMA procedure which uses a liquid ^67^Ga source with collimation.

**Results:**

Of the source-collimator configurations under investigation an optimum collimator geometry, consisting of a 5 mm thick lead disk with a diameter of 46 mm and a 5 mm central bore, was selected. The multiple window spatial registration errors obtained by the ^133^Ba method showed excellent reproducibility (standard deviation < 0.07 mm). The values were compared with the results from the NEMA procedure obtained at the same locations and showed small differences with a correlation coefficient of 0.51 (p < 0.05). In addition, the ^133^Ba point source method proved to be much easier to use. A Bland-Altman analysis showed that the ^133^Ba and the ^67^Ga Method can be used interchangeably.

**Conclusion:**

The ^133^Ba point source method measures the multiple window spatial registration error with essentially the same accuracy as the NEMA-recommended procedure, but is easier and safer to use and has the potential to replace the current standard procedure.

## Background

The ability of a gamma camera to perform quantitative dual isotope imaging depends critically on its ability to accurately position photons of different energies when imaged through different photo peak energy windows. This is particularly true when localizing small organs by subtraction scintigraphy, such as the parathyroid glands[1,2] or lung perfusion-ventilation imaging [3,4]. Poor multiple window spatial resolution (MWSR) also deteriorates spatial resolution when imaging a single radionuclide that has multiple photo peaks (e.g. ^67^Ga, ^201^Tl, ^111^In). Both the NEMA [5] and the IEC [6] standards use the same procedure to assess the accuracy of MWSR. It consists in positioning a collimated ^67^Ga source on the detector surface of the gamma camera and determining differences in the positions of the source image at different energy window settings. The procedure has two main shortcomings, one is that the collimator used is heavy (ca. 1.2 kg) and therefore potentially dangerous to the fragile detector surface. The other one is the use of a liquid ^67^Ga source which often has to be purchased specifically for this test. We developed an alternative method to assess the accuracy of multiple window spatial registration using a long-lived ^133^Ba point source collimated by a small lead collimator.

## Methods

The experiments were carried out on three camera heads of different crystal thicknesses (9.5, 15.9, and 25.4 mm) with large rectangular field of views.

**Figure 1:**
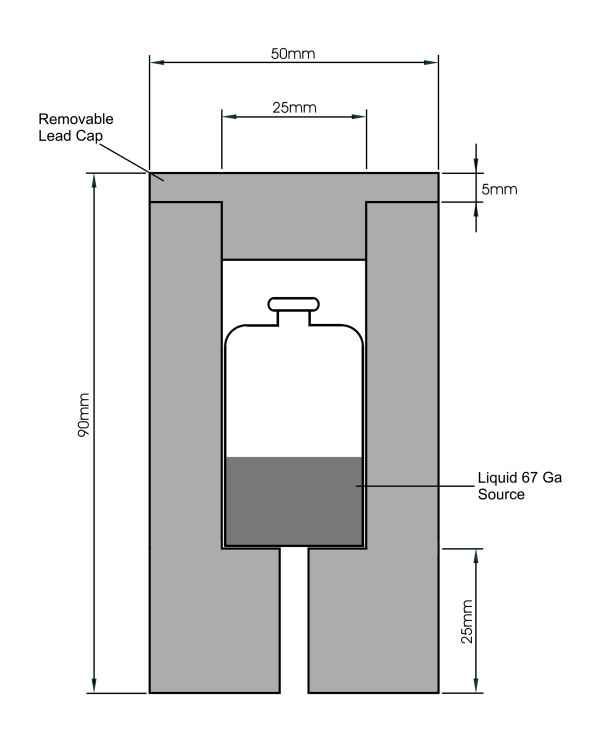
**Cylindrical lead collimator for multiple window spatial registration measurement according to **[5]** showing liquid ^67^Ga source inside.**

Reference measurements of the multiple window spatial registration error were made with a ^67^Ga source (activity at the time of measurement ca. 18 MBq). The vial containing the ^67^Ga was placed in the standard lead collimator as defined by the NEMA standard (Fig. 1) and placed at five arbitrary positions (centre, +x, -x, +y and -y) on the detector surface, with the ± x and ± y positions at about 2/3 of the distance between the centre and the respective border of the field of view. Only 5 positions were measured, compared to the NEMA recommended 9 positions. To ensure accurate and reproducible positioning, a template made of Perspex with machined recesses into which the collimator accurately fitted was used. The ^133^Ba point source is a standard commercial product (Spectrum Techniques, Oak Ridge, TN). The point source with a circular radioactive area of less than 0.2 mm diameter is embedded in the centre of a round plastic disk with a diameter of 26 mm and a total thickness of 2.7 mm. It had an activity of about 300 kBq at the time of measurements. The point source was imaged at exactly the same five positions using the same template with additional concentric recesses to fit the collimators for accurate positioning.

**Figure 2:**
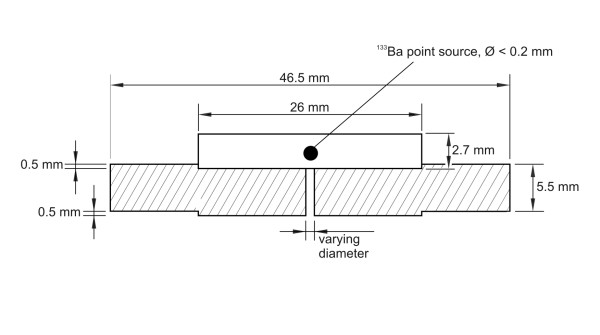
**Mini collimator made of lead showing ^133^Ba point source in plastic disc on top.** Collimator hole sizes were 2, 3, 4 and 5 mm.

The collimators for the ^133^Ba point source were made of lead with dimensions given in Fig. 2. Collimators with central bores of 2, 3, 4 and 5 mm were produced. On the upper side of the collimators was a 0.5 mm thick recess tightly fitting the point source disk. The geometrical positioning of the source was thus ensured to be accurate within 0.1 mm. The weight of a collimator was 102 g.

For each position and collimator geometry two images for the low energy photons and the high energy photons of either the ^67^Ga or ^133^Ba source were acquired for 120 s in a 512 × 512 matrix, pixel size 1.1 mm. The peak energy settings were 93 and 300 keV for ^67^Ga and 80 and 356 keV for ^133^Ba, respectively, with all energy windows set to 20%. The position of the source in an image was determined according to the NEMA algorithm by calculating the centre of gravity of the intensity within a square region with a side length of four times the full width at half maximum (FWHM) of the peak. To avoid potential errors from differences in region positioning a program written in Matlab (Version 2006a, The Mathworks Inc., Natick, MA) was developed in which the approximate position of the point source was first identified manually, followed by a fully automatic placement of the region of interest and calculations of the registration offsets. In order to identify optimum collimator dimensions, initial experiments were made using collimator bore diameters of 2, 3, 4 and 5 mm as well as the ^133^Ba source without collimation. Since the collimators with 2 and 3 mm bores were inferior to the collimators with larger holes with respect to sensitivity (see results section), the localization comparisons with the ^67^Ga values were then made with the collimators with a hole size of 4 and 5 mm and with the ^133^Ba source without collimation.

In order to facilitate the comparison of the results, the differences between low and high energy positions for the two radionuclides were displayed visually. The similarity between the locations of the two radionuclides were analyzed further by performing regression analyses using the statistical software SPSS (Version 14.0.1, SPSS Inc., Chicago, IL). Statistical significance was assumed for a p value < 0.05. A Bland-Altman analysis [7] was performed for statistically significant regressions.

Reproducibility was tested both for the ^133^Ba method using the 5 mm collimator and the ^67^Ga method by acquiring low and high energy images in one template position. For each configuration 10 measurements were carried out. Between each measurement the source with collimator was completely removed and positioned anew. Reproducibility and accuracy of the centre of the ^133^Ba source were tested by acquiring low and high energy images in one template position, for the 5 mm collimator. Eight acquisitions were performed. Between each of these acquisitions the point source was rotated by 45°.

## Results

**Figure 3:**
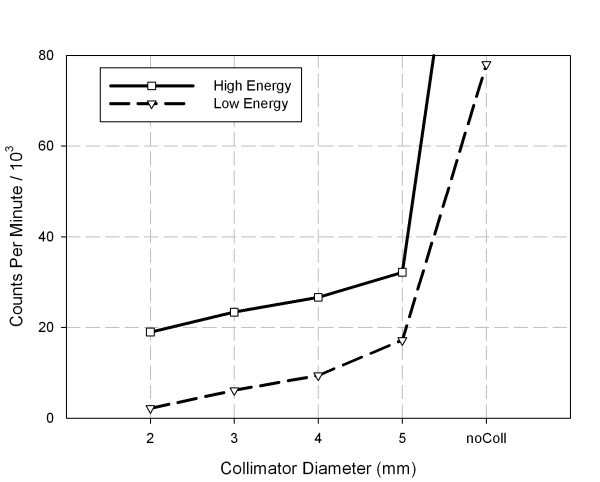
**Counts per minute in low and high energy windows for different collimator hole diameters of mini collimator.** noColl is for uncollimated ^133^Ba source. Detector thickness for results shown is 15.9 mm.

**Figure 4:**
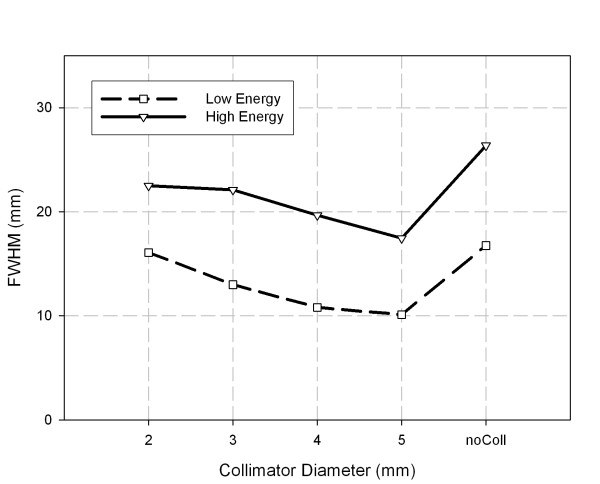
**Full width at half maximum (FWHM) of point source image for different collimator hole diameters of mini collimator.** noColl is for uncollimated ^133^Ba source. Detector thickness for results shown is 15.9 mm.

The total counts on the detector surface from the ^133^Ba source for different collimator bores (Fig. 3) show a sharp increase in the total counts for the uncollimated source, which for the high energy window amounts to an increase by a factor of 5 compared to 5 mm collimation, for the low energy window to an increase by a factor of 7.3. The count rate for the uncollimated source was larger than 100 kcps. Also, the images showed low intensity scatter radiation over the field of view of the detector, whereas scatter was negligible with collimation regardless of the hole diameter. The FWHM of the collimated point sources (Fig. 4) decreases with increasing hole size and increases again for the uncollimated source. The decrease of FWHM with increasing hole size is due to an increase of maximum count proportional to the area of the hole whereas the width of the point source image increases approximately linearly. The distinct increase of the FWHM of the uncollimated source is due to the slightly different position of the source which was in direct contact with the detector surface. The values for the full width at tenth maximum (FWTM) were approximately 3 times larger than the FWHMs and showed the same trend, especially also an increase for the uncollimated source. We decided to carry out the localization analyses because of the distinctly better sensitivity for the 4 mm, 5 mm, and the uncollimated source geometries only.

Reproducibility expressed as standard deviation of the 10 measurements was 0.07 mm for the ^133^Ba and 0.17 mm for the ^67^Ga displacement. When rotating the 133Ba source in the collimator, reproducibility expressed as standard deviation of the 8 measurements was 0.2 mm. This was due to a small displacement of the point source activity by 0.1 mm from the geometric midpoint of the circular disk containing the source. The additional error was avoided in the subsequent measurements by using always the same angular orientation of the disk with respect to the template.

**Figure 5:**
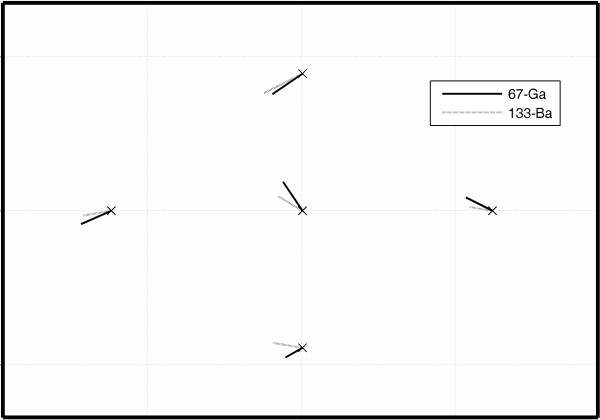
**Vector plot of differences in positions between low (marked x) and high energy positions for the nuclides ^67^Ga and ^133^Ba for a collimator hole size of 5 mm and the 9.5 mm thick detector.** For better visibility the differences are scaled by a factor of 40.

**Figure 6:**
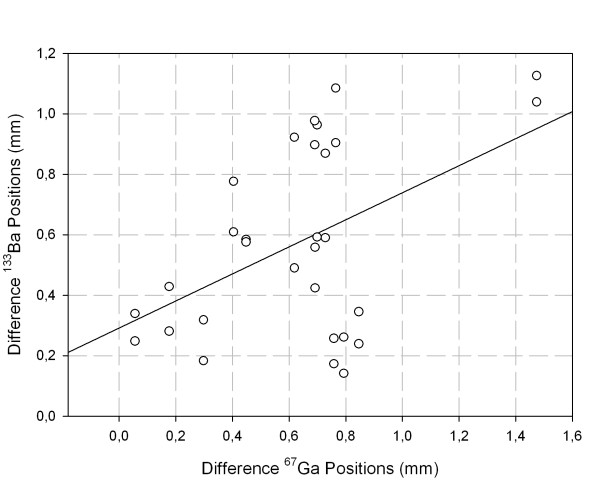
Regression for differences between low and high energy positions of the ^133^Ba point source images with 4 and 5 mm collimation on the corresponding differences for the ^67^Ga source images.

**Figure 7:**
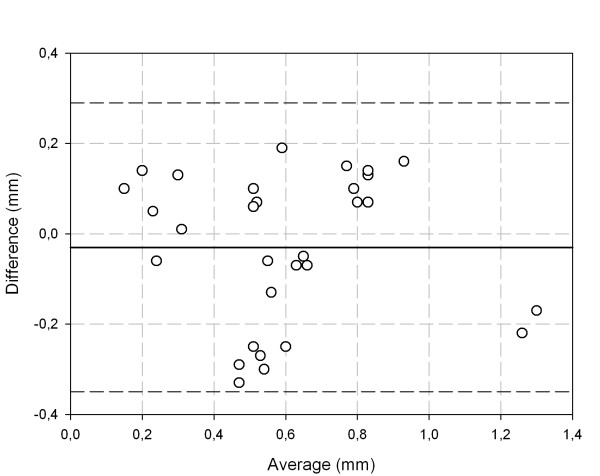
**Bland-Altman analysis of Fig. **6. The solid line indicates the mean difference between the methods, and the 95% confidence intervals for the differences are indicated by dashed lines.

Visually, the displacement between the locations for the low and the high energy windows were very similar for all detectors investigated when using the collimators with 4 mm and 5 mm bores. A representative example is shown as a vector plot in Fig. 5 for the 9.5 mm thick detector and the 5 mm bore, which show magnitude and directional differences in registration between the two nuclides. Please note that for a better display the distances are scaled up by a factor of 40. The agreement in location for the uncollimated ^133^Ba source and the ^67^Ga source is worse. The linear regression comparing the ^133^Ba displacements to the corresponding ^67^Ga displacements shows a significant correlation between the two collimated ^133^Ba source configurations and ^67^Ga (N = 30, r = 0.51, p = 0.008; Fig. 6), whereas the regression of the uncollimated ^133^Ba source on the ^67^Ga source was not significant (N = 15, r = 0.34, p = 0.23). The Bland-Altman diagram comparing the two collimated ^133^Ba source configurations and ^67^Ga shows no obvious relation between the difference and the average. The limits of agreement are -0.35 mm and 0.29 mm with the mean at -0.03 mm (Fig. 7).

The multiple window spatial registration error defined by NEMA as the maximum difference in either x- or y-coordinates between the positions at different energies is shown in Table 1. Also here, a qualitative agreement between the collimated point source configurations and the ^67^Ga measurement was found.

**Table 1:** NEMA multiple window spatial registration (MWSR) error for the 3 camera heads

	MWSR Error (mm)	
	
Detector Thickness (mm)		
	^67^Ga	^133^Ba

9.5	0.7	0.4

15.9	1.6	1.0

25.4	0.7	1.0

NEMA multiple window spatial registration (MWSR) error for the 3 camera heads, given as the maximum deviation over the 5 point source positions. The ^133^Ba MWSR errors are obtained for the 5 mm collimator hole size configuration.

## Discussion

Modern gamma cameras employ several corrections to improve non-linearity and non-uniformity. The correction parameters are obtained by tuning, carried out for the different radionuclide energies separately [8]. The basic performance parameters of a gamma camera are primarily measured with ^99m^Tc. Good accuracy of multiple window spatial resolution ensures that performance parameters such as spatial linearity or pixel calibration are not dependent on the energy (i.e. magnitude of light output). The spatial registration error of modern gamma cameras is usually specified as being below 1 mm. This limit of accuracy is acceptable for all applications of dual isotope imaging or single isotope imaging with multiple energy windows, and is achieved after tuning of the gamma camera for the particular energies in question. The proposed method for assessing the multiple windows spatial registration error has shown to give similar results as the NEMA method, but the measurement procedure is much easier and, because of the light weight collimator used with the ^133^Ba source, potentially safer to carry out. The different collimators tested show that holes of 4 and 5 mm diameter provide essentially the same results, although the better counting efficiency of the latter makes it the better choice. The Bland-Altman analysis shows that the limits of agreement between the ^133^Ba and the ^67^Ga Method are approximately one third of the usual limit for the spatial registration error of a modern gamma camera. This indicates that both methods can be used interchangeably. Although even the uncollimated ^133^Ba source gives acceptable results, the high fraction of counts outside the region of interest due to a greater divergence of the photons incident on the crystal renders the analysis more complicated since it is not possible to sum all individual point source images for joint analysis because of overlapping intensities as can be done with the collimated images. Furthermore, the source strength of the ^133^Ba source is more critical because the uncollimated count rate may easily exceed the recommended maximum count rate of 30 kcps for the measurement of performance parameters. A drawback of the method may be seen in the fact that ^133^Ba has only 2 photon energies, whereas ^67^Ga offers 3 including one close to the energy of ^99m^Tc. However, we believe it to be sufficient to measure at a low and a high energy covering the range of all commonly used radionuclide energies. This is consistent with the NEMA standard in which the low and the high energy of ^67^Ga are to be used if only 2 energy windows are available. Another advantage of the proposed method is that due to the long half-life of ^133^Ba a standard acquisition procedure including a fixed optimal acquisition time can be used which makes it easy to carry out the test routinely, such as after recalibration of the gamma camera.

## Conclusion

Multiple window spatial registration can alternatively be measured with a ^133^Ba point source and a mini-collimator weighing less than 8 percent than the standard NEMA collimator. The spatial displacement errors are quantitatively the same as those obtained using the standard NEMA procedure employing a liquid ^67^Ga source and a heavy collimator. Advantages of the alternative method are the use of a long-lived radioactive nuclide, no need for handling a liquid radioactive source, and the use of a light-weight collimator which makes the procedure much easier and potentially safer to carry out than the standard NEMA procedure. Possible disadvantages are the lack of an energy peak close to 140 keV and the need to buy and store a long lived ^133^Ba source. The ^133^Ba point source method has the potential to replace the NEMA procedure for the measurement of multiple window spatial registration errors.

## Competing interests

The authors declare that they have no competing interests.

## Authors' contributions

HB designed the study and performed the data analysis. GM, MR and PMS carried out the measurements and contributed to the preparation of the manuscript.

## Pre-publication history

The pre-publication history for this paper can be accessed here:


